# 

**Published:** 1886-12-11

**Authors:** 


### THE HOSPITAL

An Institution, Family, and Parochial Journal of Hospitals, Asylums, & all Agencies for the Care of the Sick, Criticism & News.

### CONTENTS.

**The Queen's Jubilee:** How will the Hospitals Celebrate it? - 171
**Words of Consolation.**—XI. Cheerfulness. - 172
**Hospital Administration.**—Hospital Revenues To-day and Ten Years Ago - 173
**A Hospital Impostor** - 174
**Sister Olive:** A Hospital Romance.—By W. J. Eccott 175
**Among the Out-Patients.** By One of the Crowd 178
**"The Hospital" and "The Globe."** Lord Salisbury's Article - 180
**Notes and News.**—The late Mr. O. E. Coope.—Lord Salisbury's Appeal for the Hospitals.—The Queen's Jubilee Memorials.—Miscellaneous.—Enlargement of the Bristol Eye Hospital.—Vacancies.—Competition Prizes for Orphanage Designs.—The Globe Parcel Delivery Co.—Appeals.—Sherburn Hospital and the Charity Commissioners.—The Kyrle Society.—The New Infirmary at Workington.—Aberdeen Royal Infirmary, etc. - 181
**Annotations.**—Coventry's Care for its Sick Children.—The First Medical General Election - 184
**The Guild of St. Luke.**—Sermon by the Lord Bishop of London at St. Thomas's Hospital - 184
**How to Enter the Medical Profession** - 185
**Flowers, Ferns, and Ward Decorations** - 186
**Difficult Cases.**—Creeping Paralysis in a Lady - 187
**Books, Good and Otherwise.** — Pharmacopœia of the Lock Hospital.—Medical Work and Duty, etc. - 187
**The Quilter Prizes** - 187
**The Children's Corner.**—Puzzles, Answers, etc. - 188
**The Hospital Charity Box.**—Prizes and Results - 188
**In- and Out-Patients' Hospital Diary** - 189

